# Three-Dimensional Photogrammetric Assessment of Facial Symmetry Improvement Following Botulinum Toxin Treatment in Patients with Facial Palsy: An Observational Study

**DOI:** 10.3390/jcm14207298

**Published:** 2025-10-16

**Authors:** Robin Pradel, Barbara Lerhe, Philippe Kestemont, Charlotte Helmer, Charles Savoldelli, Olina Rios

**Affiliations:** 1 Institut Universitaire de la Face et du Cou, 31 Avenue de Valombrose, 06100 Nice, France; 2 Unité de Recherche Clinique Côte d’Azur (UR2CA), 06100 Nice, France

**Keywords:** facial palsy, botulinum toxin, 3D analysis, facial symmetry, stereophotogrammetry

## Abstract

**Background/Objectives**: Facial palsy causes both functional and aesthetic impairments, with asymmetry significantly affecting quality of life. Botulinum toxin injections are increasingly used to restore facial balance by reducing contralateral hyperactivity, but outcome assessments remain largely subjective. The objective of this study was to evaluate the ability of three-dimensional (3D) stereophotogrammetry coupled with root mean square (RMS) surface analysis to objectively quantify improvements in facial symmetry following botulinum toxin treatment and to support clinicians in patient care. **Methods**: Sixteen adults with long-standing unilateral peripheral facial palsy underwent individualized botulinum toxin injections. Three-dimensional images were acquired using the Vectra H2 system before and 2–3 weeks after injection at peak efficacy. Five facial expressions (neutral, surprise, frown, Mona Lisa smile, and forced smile) were analyzed. RMS values were calculated for the whole face and facial thirds. Clinical assessment included House–Brackmann and Sunnybrook scores. **Results**: Whole-face RMS values decreased significantly after injection (1.51 ± 0.42 vs. 1.35 ± 0.43, *p* < 0.001). Improvements were observed across all thirds, most notably in the middle third. During expression, symmetry improved for all movements, with the strongest effects for surprise (d = 1.270), Mona Lisa smile (d = 0.870), and frown (d = 0.832). **Conclusions**: Three-dimensional stereophotogrammetry with RMS analysis provides an objective and reproducible method to quantify changes in facial symmetry after botulinum toxin treatment. This technique may complement clinical scoring systems and support personalized treatment planning in facial palsy patients.

## 1. Introduction

The evaluation of facial symmetry represents a key challenge in the management of patients with facial palsy. This condition, whether of peripheral or central origin, affects not only the motor and expressive functions of the face but also has a substantial impact on quality of life due to its aesthetic and social consequences [1,2,3]. In this setting, the development of effective therapeutic strategies and robust, reliable methods for their evaluation is essential.

Among the available treatment options for addressing the sequelae of facial palsy, botulinum toxin injections have progressively gained recognition as an effective tool for facial symmetrization [4,5,6,7,8,9]. By modulating the activity of the healthy, often hyperactive, side, this approach can improve facial expression and restore a degree of facial harmony. Thus, botulinum toxin treatment is not applied with a therapeutic objective, but rather to improve facial symmetry and, consequently, facial aesthetics. Nevertheless, outcome assessment still relies predominantly on clinical scales, which evaluate motor function by comparison with the healthy contralateral side and, therefore, provide only indirect information on facial symmetry, as well as subjective clinician-rated criteria, which are prone to significant observer bias and lack the sensitivity needed to detect subtle variations in movement or volume [10].

Recent advances in three-dimensional (3D) imaging, particularly stereophotogrammetric analysis, offer new opportunities in this field. This technology enables highly accurate capture of facial topography and objective quantification of three-dimensional facial movements and changes [11,12,13]. This technique relies on the acquisition of photographs using two synchronized high-resolution, high-speed cameras that capture images from different angles (stereoscopic principle), allowing the reconstruction of a three-dimensional image [14]. This technique is particularly relevant in clinical practice, as it is non-invasive, contact-free, radiation-free, and enables rapid acquisition.

A technique for evaluating facial symmetry based on the comparison of the two hemifaces has already been validated and applied to assess outcomes following facial reanimation surgery [15,16]. However, to our knowledge, this method has never been employed to assess the effects of botulinum toxin injections aimed at improving facial symmetry in patients with facial palsy.

The primary objective of the present study was to determine whether, and to what degree, this analysis system could objectively detect improvements in facial symmetry following botulinum toxin treatment. The secondary objective was to identify which parameters derived from this analysis may be the most relevant for guiding clinical interpretation and optimizing treatment outcomes in routine practice.

## 2. Materials and Methods

### 2.1. Study Population

A total of 16 adult patients were recruited during a consultation in the Department of Oral and Maxillofacial Surgery at the University Institute of the Face and Neck at Nice (31 avenue de Valombrose, 06100 Nice, France). Each patient was invited to participate in this study on a voluntary basis and signed an informed consent form after receiving prior information regarding the study procedures, the use of images and photographs for research purposes (analyses, measurements), and the possibility of their inclusion in scientific articles and publications.

This study was approved by the local Ethics Committee (approval number: 2024-016) and conducted in accordance with the principles outlined in the Declaration of Helsinki.

All patients presented with a unilateral peripheral facial palsy lasting more than one year were eligible for botulinum toxin treatment aimed at improving facial symmetry.

Inclusion criteria were as follows:

- Age > 18 years;
- Unilateral peripheral facial palsy lasting more than one year;
- Confirmed indication and treatment request for facial symmetrization with botulinum toxin injections.

Exclusion criteria were as follows:

- Age < 18 years;
- Facial scar or skin lesion that could affect facial symmetry measurements;
- History of facial surgery leading to altered facial dynamics or volumes, including facial rehabilitation techniques;
- Neurodegenerative or neuromuscular disease affecting facial movements;
- Facial palsy < 12 months’ duration (to ensure that only patients without potential for spontaneous recovery were included);
- Botulinum toxin injection to the face within the previous 6 months;
- Contraindications to botulinum toxin injection (pregnancy or breastfeeding, known hypersensitivity or allergy, neuromuscular or autoimmune disease, myasthenia gravis, and Lambert–Eaton myasthenic syndrome).

### 2.2. Facial Palsy Assessment

For each patient, the assessment of peripheral facial palsy was performed by the clinician conducting the consultation. The House–Brackmann and Sunnybrook scores [10,17,18] were calculated for each patient at the first consultation, prior to botulinum toxin injection, and again at the second consultation, after the injection. Both scores are based on a symmetry rating relative to the side contralateral to the facial palsy (the unaffected side).

### 2.3. Botulinum Toxin Injection Procedure

Following a thorough clinical examination, a personalized treatment plan was established for each patient by the clinician and recorded on a dated and signed facial diagram. The primary objective of this treatment plan was to restore facial symmetry between the right and left sides as much as possible, without adversely affecting the functional component of each muscle. The therapeutic botulinum toxin used was AbobotulinumtoxinA (Dysport^®^; IPSEN Pharma Ipsen France, 70 rue Balard, 75015, Paris, France) [19]. The doses administered ranged from 5 to 10 Speywood units per injection point. The injection sites were selected by the clinician based on the specific facial muscle activity of each patient, with the primary objective of reducing muscle activity on the healthy side.

A follow-up consultation after the injection was conducted to review the effectiveness of the treatment with the patient, confirm the absence of adverse effects, and address any additional concerns. As the maximal efficacy of botulinum toxin is observed between 14 and 28 days after injection, we decided to schedule the follow-up consultation during this period [20].

### 2.4. Image Acquisition

Three-dimensional facial images of all patients were captured using the Vectra H2 Imaging System^®^ stereophotogrammetry device (Canfield Scientific, Inc., Fairfield, NJ, USA), enabling rapid and non-invasive facial photography [13,16].

For each patient, three photographs (frontal, right three-quarter, and left three-quarter views) were required to reconstruct the final three-dimensional image of the face.

Patients were instructed to perform five different facial expressions, each engaging distinct groups of mimetic muscles:

1. Neutral: Face at rest and without expression.
2. Surprise (“raise your eyebrows”): Eyebrows elevated, appearance of frontal wrinkles, and widening of the palpebral fissures.
3. Frown (“furrow your brows”): Partial closure of the palpebral fissures and appearance of glabellar wrinkles.
4. “Mona Lisa” smile: Slight, subtle smile without tooth exposure, and mild elevation of the oral commissures.
5. Forced smile: Full, maximal, and exaggerated smile showing the teeth.

To enhance the reproducibility of the facial expressions, patients were given clear instructions, and the expected expression was demonstrated by the clinician prior to image acquisition.

In total, 15 photographs were obtained for each patient during the first consultation, prior to the botulinum toxin injection session. At the follow-up consultation, a second series of photographs was acquired following the same protocol.

### 2.5. Analysis

Using the Vectra Mirror Suite software 6.10 (Canfield Scientific Inc., Fairfield, NJ, USA), three-dimensional facial images were generated for each facial expression, yielding 10 images per patient (5 pre-injection and 5 post-injection).

**Figure 1:**
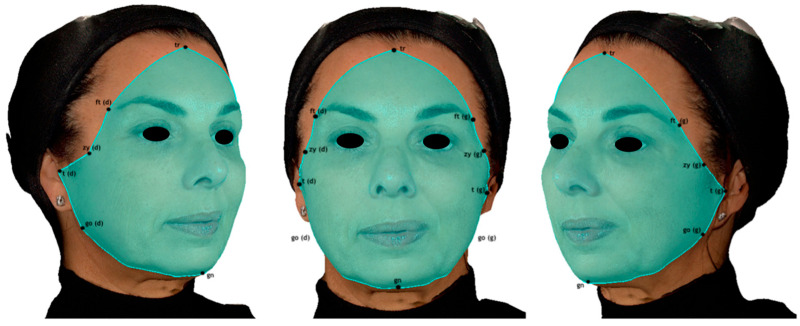
Facial area of interest (FAI), defined according to the position of the outermost anatomical landmarks.

To assess surface symmetry, a facial area of interest (FAI) was defined as the surface encompassing the following anatomical landmarks: trichion, frontotemporal points, zygion, tragion, gonion, and gnathion [15]. In this study, the FAI was defined with the objective of minimizing the inclusion of areas that could bias asymmetry quantification, such as hair and the cervical region, while ensuring that all facial structures relevant to a comprehensive morphometric analysis were included (Figure 1).

The selected surface was then used to automatically compute the plane of maximal symmetry within the software. Once the plane of maximal symmetry was obtained, each FAI was divided into right and left hemifaces. The unaffected side was mirrored, inverted, and superimposed onto the affected side, minimizing the point-to-point distance between the two superimposed surfaces.

**Figure 2:**
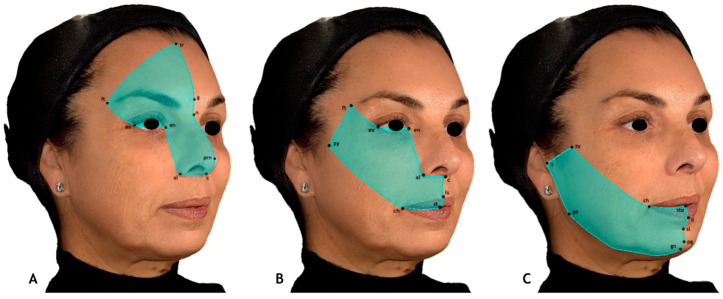
Upper third (**A**), middle third (**B**), and lower third (**C**), defined according to their respective landmarks.

Each hemiface was further divided into three different thirds: upper third, middle third, and lower third. In this analysis method, the division into thirds was based on the sensory distribution territories of the trigeminal nerve branches [15]. Each third was defined using anatomical landmarks, thereby providing standardized and reproducible selection criteria (Figure 2).

The landmarks used to define each third were as follows:

- Upper third: Trichion, glabella, pronasale, columella, alare, endocanthion, exocanthion, and frontotemporal points.
- Middle third: Endocanthion, alare, columella, subnasale, labiale superius, stomion, cheilion, zygion, frontotemporal points, and exocanthion.
- Lower third: Stomion, labiale inferius, sublabiale, pogonion, gnathion, gonion, tragion, zygion, and cheilion.

For each facial image, the root mean square (RMS) point-to-point distance between the two superimposed hemifaces was automatically calculated. The RMS distance is a statistical measure of the magnitude of differences between two surfaces, computed as the square root of the arithmetic mean of the squared point-to-point distances. In this context, it quantifies the average deviation between the mirrored unaffected hemiface and the affected hemiface, expressed in millimeters. A higher RMS value reflects a greater degree of asymmetry between the two hemifaces. A color-coded surface map was generated to represent local distance values between the two surfaces.

The calculation of RMS values has already demonstrated its reliability and accuracy in assessing facial symmetry [15]. This method has been validated and shown to be reproducible: for random errors, the selection of the facial area of interest (FAI) yields an average reproducibility coefficient (minimum detectable difference between measurements, equal to twice the standard deviation of the measurements) of 1.2 ± 0.005%, whereas for systematic errors, the mean bias value is 0.03 ± 0.001% [15]. This approach has demonstrated high interobserver reliability and reproducibility, as well as the ability to detect even subtle levels of asymmetry [16,21,22].

This procedure was applied to all facial images, both before and after botulinum toxin injection, for each facial third, resulting in a total of 320 RMS values calculated.

### 2.6. Statistical Analysis

All statistical analyses were performed using standard software and normality of distributions was verified prior to analysis. A significance level of *p* < 0.05 was considered statistically significant for all tests.

Comparisons of mean RMS values before and after botulinum toxin injection were carried out using paired Student’s *t*-tests. One-way ANOVA was employed to compare baseline RMS values between the three facial thirds (upper, middle, lower), with Tukey’s post hoc test applied when appropriate. Effect sizes were calculated using Cohen’s d to assess the magnitude of changes for each facial expression and facial third. Clinical scores (House–Brackmann and Sunnybrook) were analyzed with paired *t*-tests, and correlations between RMS values and clinical scores were explored using Spearman’s rank correlation test.

## 3. Results

A total of 16 adult patients were recruited during a consultation for facial symmetrization (Table 1).

The study population consisted of 75% women and 25% men, with a mean age of 56.8 years. Facial palsy affected the right hemiface in two-thirds of the patients.

The etiologies were as follows: idiopathic (Bell’s palsy) (50%), acoustic neuroma (25%), parotid tumor surgery (12.5%), herpes zoster oticus (6.5%), and traumatic (6.5%).

The mean interval between the two consultations was 18.5 days (SD 4.6).

**Figure 3:**
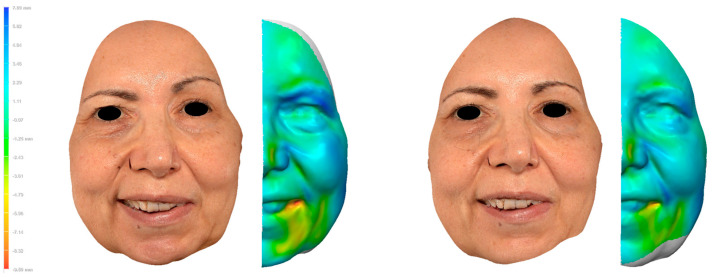
Whole-face symmetry analysis. Expression “forced smile”, before injection (**left**) and after injection (**right**). RMS before injections = 2.02 mm vs. RMS after injections = 1.52 mm. Botulinum toxin injections targeted the following muscles: right and left frontalis, right orbicularis oculi, right levator labii superioris alaeque nasi, right zygomaticus major, right and left DAO, and right and left mentalis. An improvement in symmetry is observed at the level of the oral commissures and the eyebrow position.

To understand the type of images and values obtained, a clinical case is available as an example (Figure 3).

**Figure 4:**
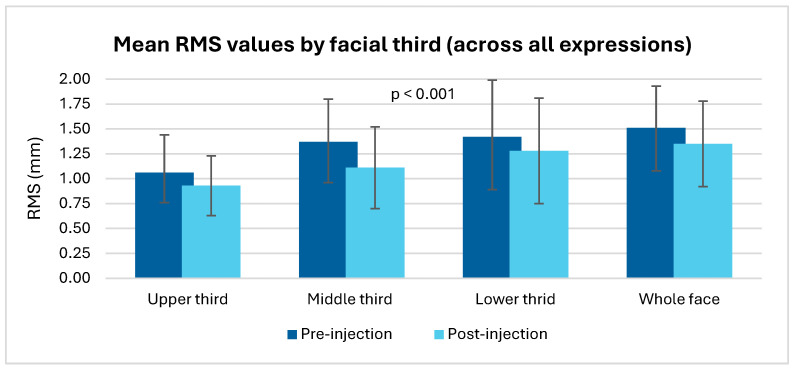
Mean RMS values by facial third (across all expressions).

Regarding our primary objective, the mean RMS value for the “whole face,” across all expressions, was 1.51 ± 0.42 before botulinum toxin injection and 1.35 ± 0.43 after injection (Table 2 and Figure 4). This reduction was statistically significant (paired Student’s *t*-test, *p* < 0.001).

The mean RMS values calculated for each facial third, before and after botulinum toxin injection, showed a significant reduction in facial asymmetry in all regions analyzed (*p* < 0.001), with the most pronounced improvement observed in the middle third (−0.26 after injection).

**Figure 5:**
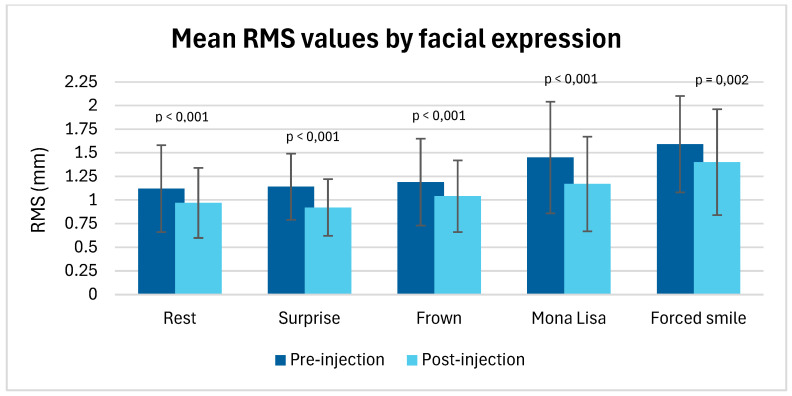
Mean RMS values by facial expression.

Mean RMS values for each facial expression also showed a significant decrease after botulinum toxin injection for all expressions evaluated (*p* < 0.05), indicating an overall improvement in facial symmetry regardless of the movement studied (Table 3 and Figure 5).

Effect size analysis (Cohen’s d) identified the facial expressions and facial thirds most sensitive to botulinum toxin injections. Among the expressions analyzed, “surprise” showed the greatest improvement after injection, with a Cohen’s d of 1.270, reflecting a very strong effect on facial symmetrization. This was followed by the “Mona Lisa” smile (d = 0.870) and “frown” (d = 0.832).

Regarding the facial thirds, the middle third demonstrated the highest sensitivity to treatment (d = 0.622), followed by the upper third (d = 0.369) and the lower third (d = 0.267).

The clinical scores of House & Brackmann and Sunnybrook (Table 4) showed a significant improvement after injections (*p* = 0.0128 and *p* = 0.0007, respectively), as did the whole-face RMS values (*p* < 0.001). However, no significant correlation was found between these types of measurements (Spearman’s correlation test).

The improvement in RMS values after injection appeared to be more pronounced in patients with severe initial facial palsy (high HB score or low Sunnybrook score). Nevertheless, the correlation analyses performed (Spearman) did not confirm this trend as statistically significant.

## 4. Discussion

The aim of our study was to determine whether this analysis system could objectively detect improvements in facial symmetry following botulinum toxin treatment, and to investigate the potential value of 3D photography and stereophotogrammetry in the evaluation and follow-up of patients with facial palsy treated with botulinum toxin injections. To this end, we assessed facial symmetry by comparing mirrored surfaces of the unaffected and affected hemifaces and calculating RMS values of point-to-point distances. This approach is well documented in the literature and has proven to be a reliable and reproducible method for analyzing facial symmetry [15,16,22,23,24,25]. It has notably been used to evaluate outcomes after facial reanimation surgery in patients with facial palsy [16]. However, to our knowledge, this method has never been applied to assess the effectiveness of facial symmetrization treatment using botulinum toxin injections in patients presenting with facial asymmetry.

In our study, the mean “whole face” RMS value, across all expressions, was 1.51 ± 0.42 before botulinum toxin injection and 1.35 ± 0.43 after injection. This reduction was statistically significant (*p* < 0.001). These results demonstrate that 3D photography combined with stereophotogrammetric analysis can objectively reveal improvements in facial symmetry after botulinum toxin injection in patients with facial palsy, using the RMS comparison method.

Moreover, the decrease in mean RMS values after injection was significant across all facial thirds as well as for each facial expression analyzed. This highlights the sensitivity of this analytical method in detecting changes in facial symmetry, even when minimal and clinically subtle, thereby reinforcing the role of 3D morphometric analysis as a valuable evaluation tool in the follow-up of patients with facial palsy.

Among the expressions analyzed, surprise showed the greatest improvement after injection, with a Cohen’s d of 1.270, indicating a very strong effect on facial symmetrization. This was followed by the Mona Lisa smile (d = 0.870) and frown (d = 0.832).

For the surprise expression, asymmetry was often moderate or even low before injection compared with other expressions, particularly those involving muscles of the middle and lower thirds. Thus, even a moderate improvement in symmetry after injection may yield a strong statistical effect, since initial variability is low.

With respect to facial thirds, the middle third demonstrated the greatest sensitivity to treatment (d = 0.622), followed by the upper third (d = 0.369) and the lower third (d = 0.267). These findings suggest that certain regions, particularly the midface, respond more markedly to botulinum toxin injections in terms of improved facial symmetry, as evidenced by decreased RMS values.

The technique of dividing the face into three thirds is not new: other authors have proposed local facial subdivisions, most often into horizontal thirds [25,26,27,28] or into a greater number of regions [23,29,30]. In this study, we followed the method described by Codari, which divides the face according to the distribution territories of the trigeminal nerve branches [15]. The selection of these three regions of distribution, corresponding to different embryological origins, is considered a subdivision that preserves the anatomical and functional integrity of the face, making it particularly suitable for morphometric analysis in patients with facial palsy. Thanks to this method and the standardized definition of facial thirds, the reproducibility of FAI selection is very high, thus overcoming the main source of intra-operator variability [15].

Regarding facial symmetry in the general population, it is known that every individual presents a certain degree of asymmetry, although it is difficult to quantify. In a cohort of 350 healthy subjects, the mean “whole face” RMS value was reported as 0.6253 ± 0.16 [21]. In another cohort of 100 healthy individuals, the mean “whole face” RMS value was 0.80 ± 0.24 mm [22]. Moreover, with respect to facial thirds, it has been demonstrated that no significant differences in symmetry exist among the three facial thirds in healthy subjects [23].

Although no significant correlation was found between the decrease in RMS values and the improvement in clinical scores (House & Brackmann and Sunnybrook [10,18]) after injections—likely due to the limited statistical power of our study—the fact that both measures improved in the same direction suggests and supports the reliability of RMS as a quantitative indicator of facial symmetry. RMS analysis may therefore serve as a complementary tool to clinical scoring systems, with which it appears to evolve consistently.

**Figure 6:**
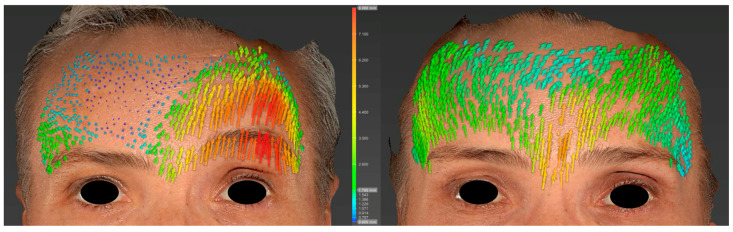
Visual comparison of cutaneous displacement vectors for the head, body, and tail of the eyebrow during the ‘surprise’ expression, before (**left**) and after botulinum toxin injection (**right**). Local skin displacement values were calculated using the automated algorithms of the Vectra Mirror Suite^®^ software and visualized according to the color and length of the vector arrows. The degree of displacement increases progressively from blue to green, yellow, orange, and finally red, with the magnitude of displacement proportional to the length of the arrows of the corresponding color.

Another complementary method of analysis is the study of cutaneous displacement vectors (Figure 6). Unlike RMS values, which provide a global and quantitative assessment of surface asymmetry, vector analysis offers a dynamic and localized evaluation of muscular activity by illustrating the amplitude and direction of soft tissue movements. By combining RMS surface asymmetry metrics with cutaneous displacement vector analysis, this integrative approach could enable the identification of the most asymmetric regions and the refinement of personalized injection strategies over time, thereby potentially optimizing facial symmetry and clinical treatment protocols.

Nonetheless, several limitations of our study and analysis protocol must be acknowledged. First, the sample size was relatively small (16 patients), although the results were highly significant (*p* < 0.001) for nearly all comparisons, with medium-to-large effect sizes (Cohen’s d 0.5–1.20), suggesting that the statistical power was likely sufficient. This study was designed as a preliminary observational study to provide pilot data, which will be extended in future research with larger cohorts. The limited sample size can also be explained by the rarity of eligible patients presenting with facial palsy requiring botulinum toxin treatment, suitable for 3D stereophotogrammetric evaluation, and willing to consent to 3D photographic acquisition.

Second, it is difficult to guarantee perfect reproducibility of facial expressions between the two consultations, as the intensity or quality of expressions may vary from one session to another (e.g., a broader smile at the second acquisition), potentially altering symmetry measurements and limiting direct comparability.

Third, a fully homogeneous analysis between patients is challenging, since injection protocols are individualized and adapted to each patient’s muscle contraction patterns. This inter-individual variability limits strictly standardized comparisons across subjects. To address this heterogeneity, the injected muscles and the facial thirds involved were systematically documented for each patient, allowing statistical analyses to be stratified by the regions treated, thereby ensuring consistent and targeted interpretation of the effects of botulinum toxin on facial symmetry.

It should also be noted that the use of stereophotogrammetric analysis software has a non-negligible learning curve, requiring progressive familiarization with measurement tools to ensure reliable and reproducible analyses.

Finally, this type of analysis quantifies global facial asymmetry but does not allow a precise distinction of the contribution of different components of facial palsy, such as loss of tone on the affected side, compensatory hyperactivity on the healthy side, or the presence of synkinesis. Clinical examination, therefore, remains essential to identify these elements and understand their role in the mechanisms of facial asymmetry.

Beyond demonstrating a significant improvement in facial symmetry after botulinum toxin injection, the results of this study open concrete clinical perspectives for personalized management of patients with facial palsy. Stereophotogrammetric analysis provides objective, reproducible, and longitudinally comparable data. These measurements not only assess treatment effects but can also guide therapeutic strategy by precisely identifying the most asymmetric regions, the most active muscles, and compensatory areas. Moreover, it could allow enhanced patient education, using interactive and intuitive 3D images. This visual support helps explain treatment goals clearly, illustrate expected outcomes, and visualize the evolution of facial symmetry after injection in a simple and understandable way, thereby improving patient understanding and adherence to the treatment plan.

## 5. Conclusions

Our study confirmed the relevance and reliability of the RMS analysis method in a clinical setting, demonstrating a significant improvement in facial symmetry following botulinum toxin treatment. Beyond the global evaluation of overall facial balance, this approach allows for a more detailed and region-specific assessment, highlighting the areas most affected by asymmetry. Such targeted analysis not only enhances the understanding of each patient’s individual facial dynamics but also supports a more personalized therapeutic strategy. Moreover, the method provides robust and reproducible quantitative data, which facilitates objective before-and-after comparisons of injections, strengthens the evaluation of treatment efficacy, and may serve as a valuable tool for both clinical practice and future research.

## Figures and Tables

**Table 1:** Study population characteristics.

Patient	Age	Sex	Side of Palsy	Etiology
1	43	M	Right	Parotid tumor
2	88	F	Right	Acoustic neuroma
3	60	F	Left	Acoustic neuroma
4	62	M	Right	Bell’s palsy
5	50	F	Left	Parotid tumor
6	41	F	Right	Bell’s palsy
7	54	F	Left	Bell’s palsy
8	64	F	Right	Herpes zoster oticus
9	46	F	Right	Bell’s palsy
10	45	F	Right	Bell’s palsy
11	65	F	Left	Acoustic neuroma
12	44	F	Left	Acoustic neuroma
13	72	M	Left	Bell’s palsy
14	57	M	Right	Bell’s palsy
15	70	F	Right	Trauma
16	47	F	Right	Bell’s palsy

**Table 2:** Mean RMS values by facial third (across all expressions).

Facial Region	Mean RMS Values (Pre-Injection)	Mean RMS Values (Post-Injection)	*p*-Value	Effect Size (Cohen’s d)
Upper third	1.06 (0.38)	0.93 (0.30)	<0.001	0.369
Middle third	1.37 (0.43)	1.11 (0.41)	<0.001	0.622
Lower third	1.42 (0.57)	1.28 (0.53)	<0.001	0.267
Whole face	1.51 (0.42)	1.35 (0.43)	<0.001	0.381

**Table 3:** Mean RMS values by facial expression.

Expression	Mean RMS Values (Pre-Injection)	Mean RMS Values (Post-Injection)	*p*-Value	Effect Size (Cohen’s d)
Rest	1.12 (0.46)	0.97 (0.37)	*p* < 0.001	0.613
Surprise	1.14 (0.35)	0.92 (0.3)	*p* < 0.001	1.270
Frown	1.19 (0.46)	1.04 (0.38)	*p* < 0.001	0.832
Mona Lisa smile	1.45 (0.59)	1.17 (0.50)	*p* < 0.001	0.870
Forced smile	1.59 (0.51)	1.40 (0.56)	*p* = 0.02	0.496

**Table 4:** House & Brackmann and Sunnybrook scores pre- and post-injection.

Patient	House & Brackmann (Pre-Injection)	House & Brackmann (Post-Injection)	Sunnybrook (Pre-Injection)	Sunnybrook (Post-Injection)
1	4	3	34	49
2	5	3	15	58
3	5	4	14	34
4	4	3	43	62
5	2	1	85	96
6	4	3	33	58
7	4	3	37	66
8	3	3	50	68
9	5	4	25	29
10	5	5	15	29
11	4	6	13	29
12	4	4	32	31
13	3	3	48	51
14	4	3	39	45
15	3	2	55	83
16	4	3	40	60

## Data Availability

The raw data supporting the conclusions of this article will be made available by the authors on request.
